# Radio-pathomic mapping model generated using annotations from five pathologists reliably distinguishes high-grade prostate cancer

**DOI:** 10.1117/1.JMI.7.5.054501

**Published:** 2020-09-09

**Authors:** Sean D. McGarry, John D. Bukowy, Kenneth A. Iczkowski, Allison K. Lowman, Michael Brehler, Samuel Bobholz, Andrew Nencka, Alex Barrington, Kenneth Jacobsohn, Jackson Unteriner, Petar Duvnjak, Michael Griffin, Mark Hohenwalter, Tucker Keuter, Wei Huang, Tatjana Antic, Gladell Paner, Watchareepohn Palangmonthip, Anjishnu Banerjee, Peter S. LaViolette

**Affiliations:** a Medical College of Wisconsin, Department of Biophysics, Milwaukee, Wisconsin, United States; b Medical College of Wisconsin, Department of Radiology, Milwaukee, Wisconsin, United States; c Medical College of Wisconsin, Department of Pathology, Milwaukee, Wisconsin, United States; d Medical College of Wisconsin, Department of Urological Surgery, Milwaukee, Wisconsin, United States; e Medical College of Wisconsin, Department of Biostatistics, Milwaukee, Wisconsin, United States; f University of Wisconsin–Madison, Department of Pathology, Madison, Wisconsin, United States; g University of Chicago, Department of Pathology, Chicago, Illinois, United States; h Chiang Mai University, Department of Pathology, Faculty of Medicine, Chiang Mai, Thailand; i Medical College of Wisconsin, Department of Biomedical Engineering, Milwaukee, Wisconsin, United States

**Keywords:** prostate cancer, machine learning, magnetic resonance imaging, rad-path

## Abstract

**Purpose:** Our study predictively maps epithelium density in magnetic resonance imaging (MRI) space while varying the ground truth labels provided by five pathologists to quantify the downstream effects of interobserver variability.

**Approach:** Clinical imaging and postsurgical tissue from 48 recruited prospective patients were used in our study. Tissue was sliced to match the MRI orientation and whole-mount slides were stained and digitized. Data from 28 patients (n=33 slides) were sent to five pathologists to be annotated. Slides from the remaining 20 patients (n=123 slides) were annotated by one of the five pathologists. Interpathologist variability was measured using Krippendorff’s alpha. Pathologist-specific radiopathomic mapping models were trained using a partial least-squares regression using MRI values to predict epithelium density, a known marker for disease severity. An analysis of variance characterized intermodel means difference in epithelium density. A consensus model was created and evaluated using a receiver operator characteristic classifying high grade versus low grade and benign, and was statistically compared to apparent diffusion coefficient (ADC).

**Results:** Interobserver variability ranged from low to acceptable agreement (0.31 to 0.69). There was a statistically significant difference in mean predicted epithelium density values (p<0.001) between the five models. The consensus model outperformed ADC (areas under the curve = 0.80 and 0.71, respectively, p<0.05).

**Conclusion:** We demonstrate that radiopathomic maps of epithelium density are sensitive to the pathologist annotating the dataset; however, it is unclear if these differences are clinically significant. The consensus model produced the best maps, matched the performance of the best individual model, and outperformed ADC.

## 1 Introduction

Despite being the most commonly diagnosed noncutaneous cancer in men, improved prostate cancer screening and aggressive therapies have led to a high five-year overall survival rate.^1^ There is still substitutional morbidity associated with overtreatment of indolent disease due to the semi-invasive nature of the current diagnostic paradigm, the 12-core transrectal ultrasound-guided biopsy.^2^ As such, there has been a growing interest in recent years for developing improved strategies for differentiating aggressive from indolent prostate cancer noninvasively.

Tumor is graded according to the Gleason pattern scale and assigned a score corresponding to the Gleason grades of the two most predominant patterns, which are then used to classify patients into five grade groups (GG) that predict prognosis.^3^ Patients with clinically significant prostate cancer (GG≥2, tumor volume≥0.5  ml, or stage≥T3) are often treated with definitive therapies (i.e., radical prostatectomy or radiation), while those with low-risk disease may be managed conservatively with active surveillance.

Accurate biopsy grading is critical for accurate staging and risk stratification, as the GG assigned at biopsy determines how aggressively a patient’s cancer will be treated. There is a growing body of literature quantifying interuser variability in pathology. In breast cancer, prior studies have shown a high degree of concordance at the extremes of a spectrum of a disease, with the highest levels of disagreement in the center of the spectrum.^4^ Similar results have been found in prostate cancer. Al-Maghrabi et al.^5^ found 100% agreement in Gleason 8 biopsy cores, but 54% and 61% for Gleason 5 to 6 (not clinically significant) and Gleason 7 (clinically significant), respectively. Other studies report similar findings, with moderate to high agreement between observers.^6^^–^^8^

Recent publications have increasingly adopted machine learning and deep learning approaches as a means to bridge the gap between radiology and pathology. In prostate cancer pathology, Karimi et al.^9^ recently achieved 86% accuracy in differentiating high-grade from low-grade pathology. Other recent studies have automated biopsy grading^10^ and annotating whole-mount slides.^11^ In prostate cancer radiology, several studies have demonstrated the ability to predict histological characteristics based on magnetic resonance imaging (MRI);^12^^–^^14^ additionally, radiomics-based approaches have been successfully implemented for risk stratification^15^ and disease characterization.^16^ Imaging focused algorithms have not yet been adopted clinically due to the lack of availability of large validation datasets and differing imaging protocols between treatment centers.^17^

The downstream effects of varying labels in pathological annotation have not been previously studied. While scanner and protocol differences are known to cause variability between sites, it is unknown whether variation in pathologists between sites causes substantial changes to the output of rad-path algorithms, which could potentially hinder intersite reproducibility. This study evaluates the performance of a previously published rad-path algorithm, radiopathomic mapping (RPM) of epithelium density,^18^ while varying the pathologist annotating the training dataset. We tested the following hypotheses: the annotations from the varying pathologists are equivalent; models trained on different annotations predict the same epithelium density values; and a consensus model outperforms apparent diffusion coefficient (ADC) with respect to classifying high-grade tumors.

## 2 Methods

### 2.1 Study Population

Data from 48 prospective and consecutively recruited patients were analyzed for this institutional review board (IRB)-approved study. A subset of this subject cohort was previously used to develop the RPM method.^18^ Written consent was obtained by a clinical coordinator. Patients scheduled for both a multiparametric (MP) MRI with endorectal coil and radical prostatectomy were considered for inclusion in this study. Patient age ranged from 42 to 72 years (mean 60). Data were collected between 2014 and 2017.

### 2.2 Imaging

Clinical imaging was acquired using a single 3T scanner (Discovery MR750, General Electric, Waukesha, WI) with an endorectal coil (Medrad Prostate eCoil, Bayer Healthcare, Warrendale, PA). The MP protocol included T2 weighted imaging, dynamic contrast-enhanced imaging, and field of view optimized and constrained single-shot diffusion with 10 b-values (0, 10, 25, 50, 80, 100, 200, 500, 1000, and $2000\;{s/\mathrm{mm}}^{2}$). The imaging was processed as previously published.^18^^,^^19^ The images used to generate the radiopathomic maps were intensity normalized T2, ADC (b=0 and $1000\;{s/\mathrm{mm}}^{2}$), ADC (b=1000 and $2000\;{s/\mathrm{mm}}^{2}$), ADC (b=500 and $2000\;{s/\mathrm{mm}}^{2}$), b=0, and a T1 subtraction map.^18^^–^^20^

### 2.3 Tissue Processing

Robotic prostatectomy was performed using a da Vinci system (Intuitive Surgical, Sunnyvale, California) by a single fellowship-trained surgeon (K.J.). Prostate samples were fixed in formalin overnight and sectioned using custom 3D-printed slicing jigs created to match the orientation and slice thickness of the T2 weighted image.^19^^,^^21^ Whole-mount tissue sections were paraffin embedded, hematoxylin and eosin (H&E) stained, digitally scanned at 40× using a microscope with an automated stage (Nikon Metrology, Brighton, Michigan), and downsampled to 8× resolution prior to annotation.

### 2.4 Pathologic Annotation

**Fig. 1:**
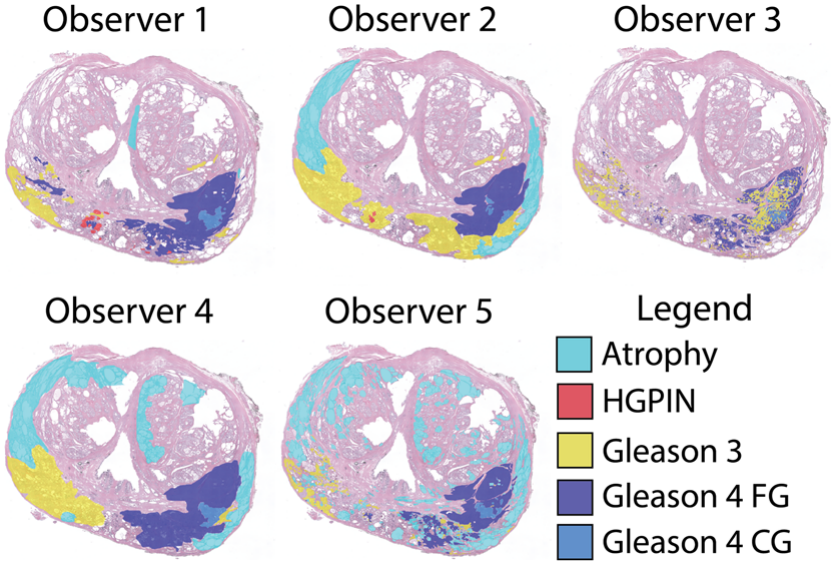
Whole-mount H&E-stained prostate slide annotated by five pathologists. While all observers have noted lesions in the left and right peripheral zone, the lesion size and grade vary slightly between observers. The annotation style differs between observers; in particular, how fine-grained the annotation is (observer 3 versus observer 4), and whether they have chosen to explicitly define atrophy. HGPIN, high-grade prostatic intraepithelial neoplasia; FG, fused gland; and CG, cribriform gland.

Two datasets of whole-mount slides were used in this study: the single annotation (SA) set annotated by one pathologist (K.A.I.), and the multiannotation (MA) set annotated by five pathologists (K.A.I., W.P., T.A., G.P., and W.H.). The SA dataset consisted of 123 slides taken from 20 patients, and the MA dataset consisted of 33 slides from 28 separate, nonoverlapping patients. The number of slides in the MA dataset was selected such that it would produce sufficient data to train a stable RPM algorithm. The SA dataset accounted for slides from all remaining patients in our dataset not already included in MA. Slides were annotated using a stylus on a Microsoft Surface with a preloaded color palette for ease of use. Both datasets were first annotated by K.A.I., and the MA dataset was selected as a subset encompassing all unique labels. Slides were annotated according to the Gleason pattern scale, including high-grade prostatic intraepithelial neoplasia (HGPIN), benign atrophy, Gleason pattern 3 (G3), Gleason pattern 4 fused gland (FG) and cribriform gland (CG), and Gleason pattern 5. A slide with annotations from all pathologists can be seen in Fig. 1.

### 2.5 Segmentation and Co-Registration

The high-resolution pathology was segmented pixel-wise into lumen, epithelium, and other tissue using a morphological algorithm previously published.^18^ The slides were then downsampled and a control point was warped to match the corresponding axial slice on the clinical T2 weighted image as previously published using custom-developed code.^19^^,^^20^ The computational segmentation and pathologist annotations were then aligned using the calculated transform.

### 2.6 Experiment 1: Pathologist Variability

**Fig. 2:**
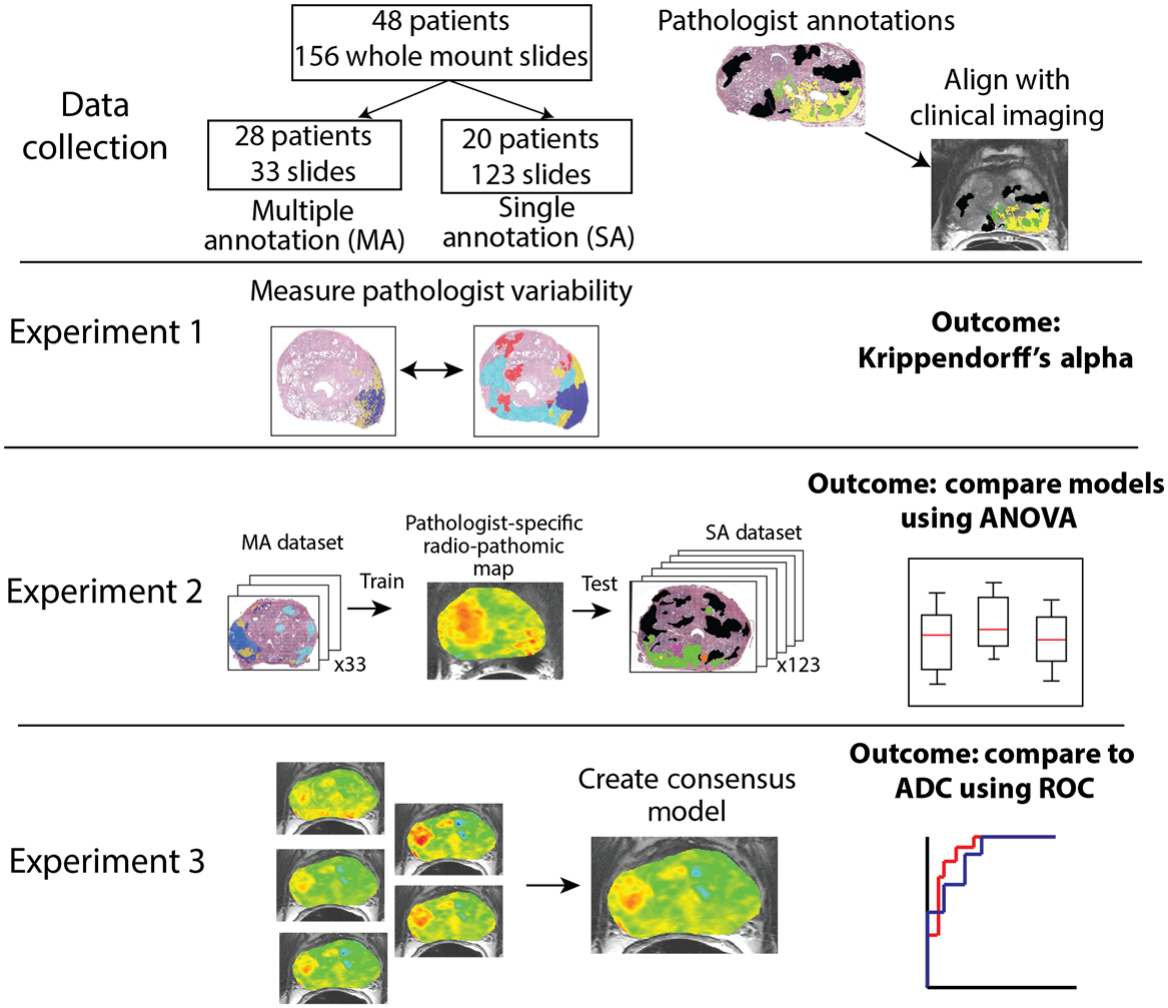
Experimental set up and outcomes of this study. In experiment 1, whole-mount annotations were compared between pathologists pair-wise using Krippendorff’s alpha. Experiment 2 trains pathologist-specific RPM models and compares the mean epithelium density values on a held-out test set. Experiment 3 combines the pathologist-specific models into a consensus model and compares the area under the ROC curve to ADC classifying high-grade tumors versus low-grade and benign regions.

The workflow and outcomes of this study can be seen in Fig. 2. Interobserver variability was measured using Krippendorff’s alpha. Pathological annotations were grouped into three categories: unlabeled, low grade, and high grade. Krippendorff’s alpha measures interobserver variability similar to Cohen’s kappa,^22^ but is better suited for cases with multiple observers and multiple, ordinal categories. Measures of interobserver variability assume identical boundaries; to account for the varying boundaries between observers, the test was repeated once for each pathologist, measuring interobserver variability using different observers’ annotations each iteration. The outcome is reported as a Krippendorff’s alpha coefficient with bootstrapped 95% confidence intervals.

### 2.7 Experiment 2: Generation of Pathologist-Specific Radiopathomic Maps

Radiopathomic maps were generated by training a partial least-squares regression algorithm with one latent variable. The clinical imaging values served as the input features to predict the epithelium density values calculated on the whole-mount pathology. The training dataset was generated lesion-wise using the median value in a pathologist-drawn region of interest (ROI). Lesions >200  voxels ($0.1\;{\mathrm{cm}}^{2}$) in plane were included. In the SA dataset, regions of benign atrophy were used as controls to capture a full disease spectrum. In the MA dataset, however, the amount of atrophy annotated varied dramatically between observers. An unlabeled consensus was used to standardize the nondiseased regions in the MA dataset and encompass a full spectrum of disease and healthy tissue. To create these masks, all voxels labeled as Gleason pattern 3 or higher (G3+) by any pathologist were subtracted from the prostate mask. Four equally sized ROIs were then generated from the remaining unlabeled area and added to the dataset as unlabeled consensus tissue.

Models were trained on 33 slides from the MA dataset and applied to the SA dataset. Additionally, a consensus model was generated by averaging the predicted values from the five models. A repeated measures analysis of variance (ANOVA) was performed to test the hypothesis that the mean predicted epithelium value was identical for each of the five models. Tukey’s honest significant difference was applied to identify which models differed.

### 2.8 Experiment 3: Consensus Model

A consensus model was created by averaging the outputs of the pathologist-specific models. The model was evaluated on the SA dataset using an empirical receiver operator characteristic (ROC) curve; the area under the curve (AUC) served as the figure of merit. The AUC evaluated the model’s ability to differentiate high-grade lesions (G4+) from benign and low-grade (G3) regions. Bootstrapped 95% confidence intervals were calculated for observation on the AUC using 500 bootstrap iterations. ADC ($b=0,1000\;{s/\mathrm{mm}}^{2}$) was evaluated under the same conditions. The input for this test was the predicted epithelium density value from the model. A t-test equivalent was calculated between the ROC from the consensus model and the curve for ADC as described by Hanley and McNeil,^23^ and a z-score was calculated using the following equation:

$$z=\frac{A_{1}-A_{2}}{\sqrt{SE_{1}^{2}-SE_{2}^{2}-2rSE_{1}SE_{2}}},$$ (1)

where $A_{1}$ and $A_{2}$ are the areas under the ROC for the two models to be compared, ${\mathrm{SE}}_{1}$ and ${\mathrm{SE}}_{2}$ are the standard error (SE) terms for the respective models, and r is found using a lookup table provided by Hanley and McNeil based on the average AUC and the Kendall–Tau correlation coefficient between the two models calculated separately on the positive and negative examples.

The SE is calculated as follows:

$$\mathrm{SE}=\frac{\sqrt{\mathrm{AUC}*(1-\mathrm{AUC})+(N_{1}-1)*(Q_{1}-{\mathrm{AUC}}^{2})+(N_{2}-1)*(Q_{2}-{\mathrm{AUC}}^{2})}}{N_{1}*N_{2}},$$ (2)

where $N_{1}$ is the number of positive examples and $N_{2}$ is the number of negative examples. $Q_{1}$ and $Q_{2}$ can be calculated using Eqs. (3) and (4)

$$Q_{1}=\frac{\mathrm{AUC}}{\left(2-\mathrm{AUC}\right)},$$ (3)

$$Q_{2}=\frac{2{*\mathrm{AUC}}^{2}}{1+\mathrm{AUC}}.$$ (4)

A p-value is calculated from the area under the z distribution more extreme than the observed z value. Significance was set to the 0.05 level. The AUC and bootstrapped 95% confidence intervals were calculated on all five models for the purpose of observation.

## 3 Results

### 3.1 Experiment 1: Pathologist Variability

Interobserver agreement is shown in Table 1. We found moderate agreement (mean = 0.50) with substantial variability depending on which observer’s annotations were used.

**Table 1:** Krippendorff’s alpha, each value is calculated considering all observers within one observer’s ROI.

Observer	Krippendorff’s alpha (95% CI)
Observer 1	0.46 (0.30 to 0.61)
Observer 2	0.31 (0.18 to 0.42)
Observer 3	0.69 (0.55 to 0.80)
Observer 4	0.49 (0.35 to 0.60)
Observer 5	0.57 (0.41 to 0.70)

### 3.2 Experiment 2: Generation of Pathologist-Specific Radiopathomic Maps

**Fig. 3:**
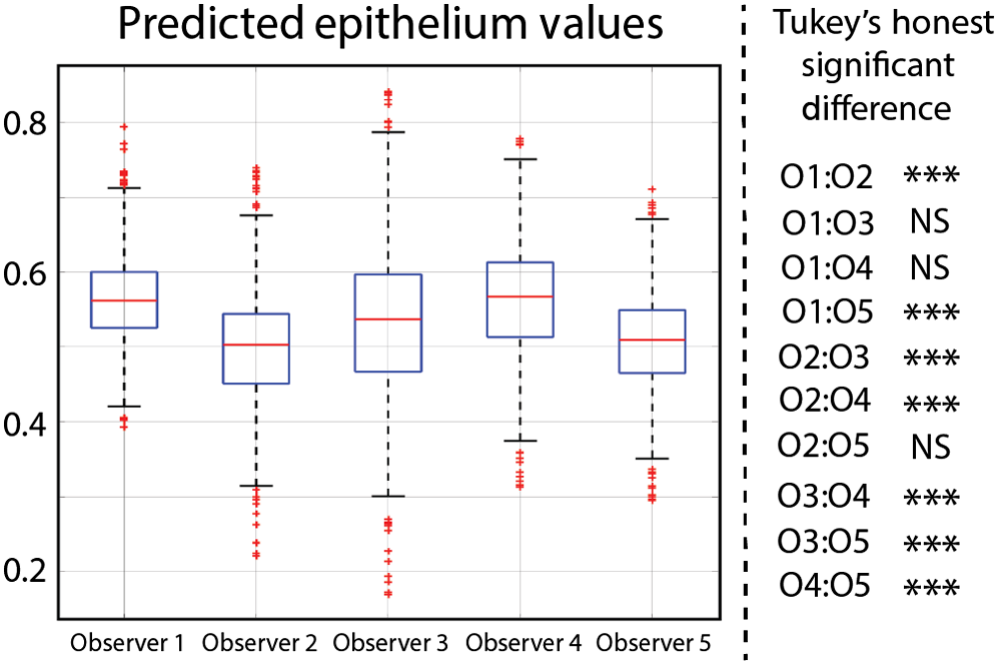
ANOVA analysis comparing the mean predicted epithelium values for each model. Pair-wise comparisons were performed using Tukey’s honest significant difference.

The results of experiment 2 can be seen in Fig. 3. There was a statistically significant difference in the mean predicted epithelium values between the five models (p<0.001). Of the 10 pair-wise combinations, seven are statistically different. Comparisons between model 1 and model 4, model 1 and model 3, and model 2 and model 5 are not statistically significant.

### 3.3 Experiment 3: Consensus Model

**Fig. 4:**
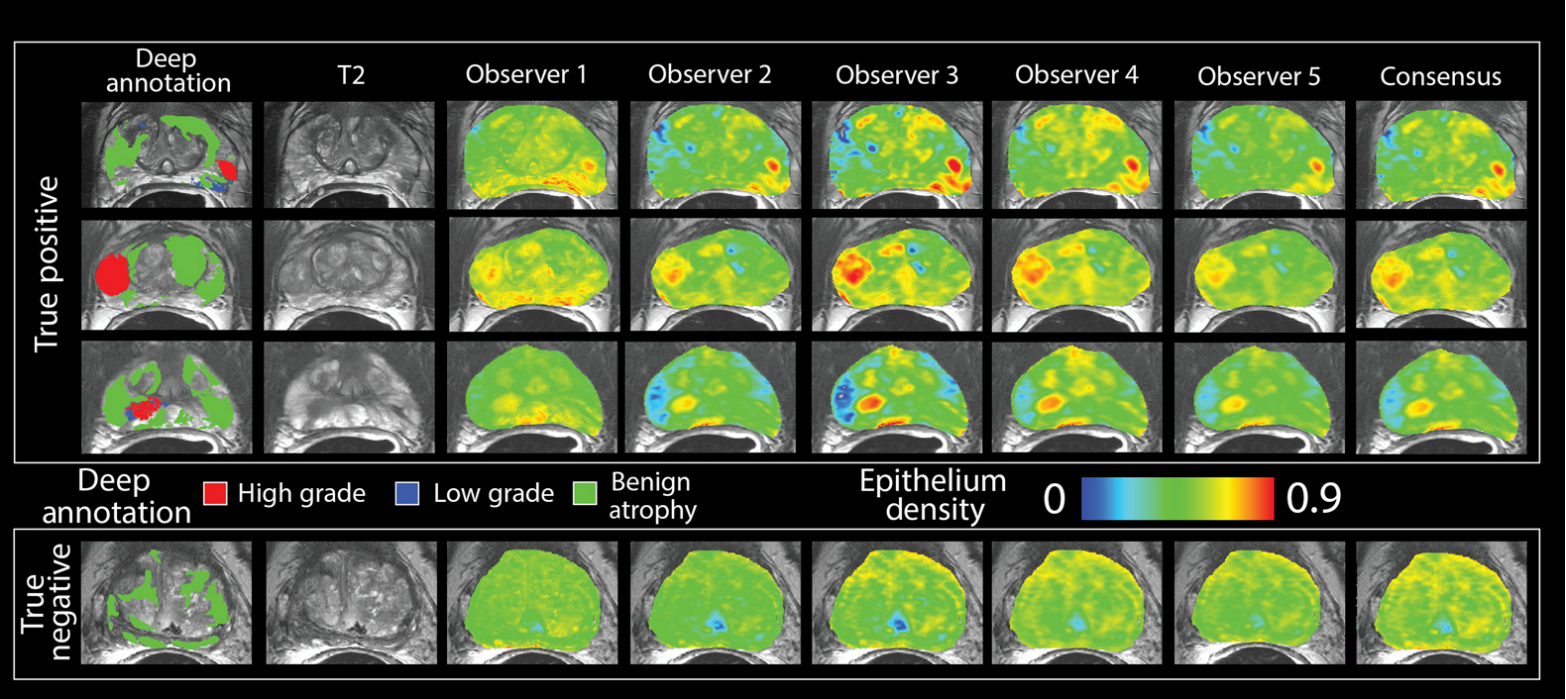
(a) Deep annotation showing a single observer’s annotation of atrophy, low-grade, and high-grade prostate cancer overlaid on the T2. (b) Voxel-wise predictions of epithelium density in MRI space in three true positive cases (top) and one true negative case (bottom). Susceptibility to image noise and signal intensity varies across observers. The consensus model is generated by averaging the maps from the five observers.

**Fig. 5:**
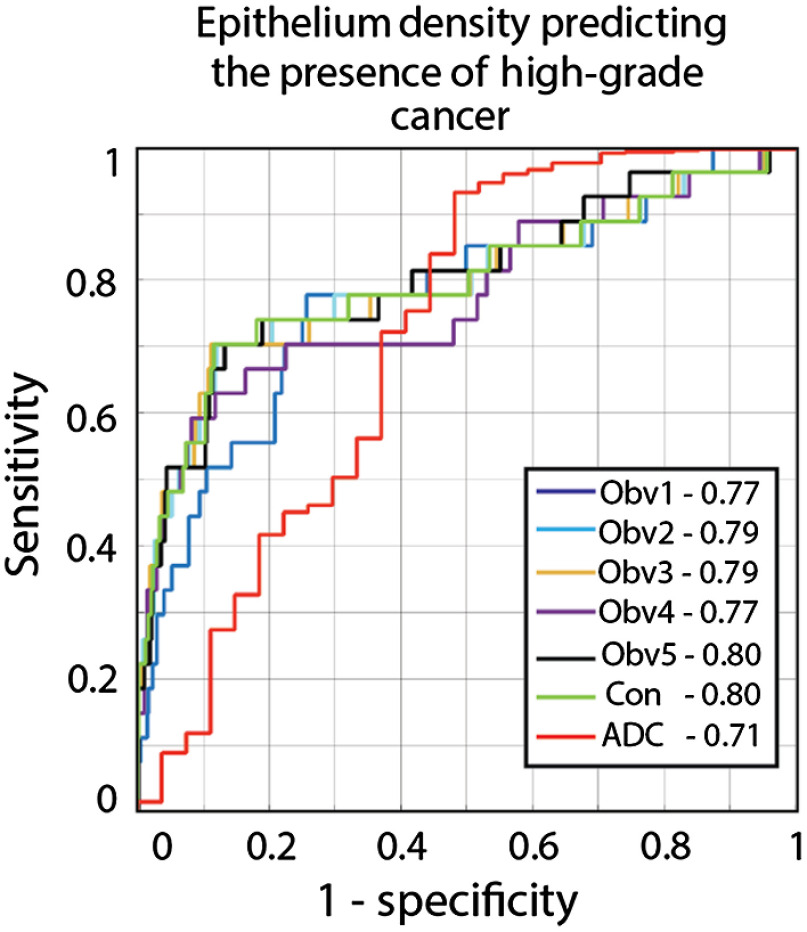
The performance of the five RPM models, consensus model, and ADC were evaluated using an empirical ROC curve. The classification task was identifying high-grade tumors from low-grade and benign regions. RPM models consistently outperform ADC alone and the consensus model matches the AUC of the top pathologist. The consensus model statistically outperforms ADC.

Representative images showing the output of the RPM models and the consensus model are shown in Fig. 4. The AUC quantifying how effectively predicted epithelium density stratified high-grade cancer from low-grade and benign regions ranged from 0.77 to 0.80, with the consensus model at 0.80, and the results are shown in Fig. 5. ADC analyzed under the same conditions produced an AUC of 0.71. Bootstrapped 95% confidence intervals can be seen in Table 2. The consensus model outperforms ADC (p<0.05).

**Table 2:** Area under the ROC curve and bootstrapped confidence intervals. Results calculated on the SA dataset.

Condition	AUC (95% CI)
Observer 1	0.77 (0.66 to 0.86)
Observer 2	0.79 (0.67 to 0.88)
Observer 3	0.79 (0.67 to 0.90)
Observer 4	0.77 (0.65 to 0.88)
Observer 5	0.80 (0.65 to 0.88)
Consensus	0.80 (0.66 to 0.90)
ADC	0.71 (0.57 to 0.83)

## 4 Discussion

This study demonstrates that radiopathomic maps of epithelium density are sensitive to the pathologist annotating the training dataset. Five models trained on the same data annotated by five different pathologists performed better than ADC alone, but with some variability between models. There is a statistically significant difference in the mean epithelium values predicted by each model; however, the number of lesions in the test dataset is large and thus a significant result can be produced from a relatively small effect size. Further studies are required to determine if this difference is clinically significant. The five individual models can be combined to create a consensus model with an area under the ROC curve statistically greater than that of ADC analyzed under the same conditions.

Our analysis of pathologist-annotated regions of cancer resulted in moderate agreement. Other studies have noted moderate agreement between pathologists, with the focus of disagreement occurring around the G3 to G4 boundary. Previous studies examining pathologist variability have used biopsy cores rather than whole-mount slides and use some variant of Gleason grade grouping to reduce the number of categories examined.^7^^,^^24^ Several factors influence the differences between the prior studies and our present study. The observers were presented with whole-mount slides rather than biopsy cores, and as a result, both boundary and disease severity could vary. The slides were digitized, downsampled, and viewed on a tablet rather than under a microscope. The resolution of the digital images likely decreased interobserver agreement; however, it was infeasible to store full resolution images on the tablets distributed to the observers for annotation. Furthermore, the smaller each individual ROI becomes the more difficult it is to use in MRI space. More research is necessary to quantify the effects of digital slide resolution on annotation repeatability.

Annotation style may also directly influence interobserver agreement. For example, observer 3’s annotation style was focused on annotating individual glands as separate ROIs, while the others focused more on annotating larger regions containing many glands. While observer 3’s style is highly useful for a study in pathology, small ROIs are more susceptible to imperfect alignment caused by the difference in scale between clinical imaging and pathology and thus were also likely to be excluded from the analysis. There may have been less variability in the performance of the five models if the annotation style was consistent between the pathologists; future studies should consider careful instruction for how annotations are generated to facilitate more generalizable results.

In the last decade, a number of other groups have written algorithms capable of predicting Gleason grade or a histomorphometric characteristic correlated with cancer severity from clinical MRI in prostate cancer.^15^^–^^17^^,^^25^^–^^29^ Radiomics-based approaches remain the most popular in image space due to the lack of availability of large, pathologically confirmed image datasets required to validate a deep learning approach.^30^^–^^32^ The use of aligned whole-mount source material is becoming preferable to biopsies, as the whole-mounts provide more information. Reported ROC results vary from 0.77 to 0.99 comparing cancer with benign tissue. While it has been reported that cancers with a total volume $>0.5\;{\mathrm{cm}}^{2}$ on whole-mount slides are more likely to be significant,^33^ we have found that our technique is sensitive to lesions as small as $0.1\;{\mathrm{cm}}^{2}$. No standard value for lesion exclusion exists in whole-mount rad-path studies, which may have implications for the comparison of ROC results between rad-path studies in prostate cancer. Chatterjee et al.^25^ reported excluding lesions smaller than $5\times 5\;{\mathrm{mm}}^{2}$ from their analysis, but not all studies explicitly report a value. Future studies should explicitly define an acceptable cut-off range to better facilitate comparison between techniques and between sites.

The outcome of this study was intentionally chosen to be epithelium density rather than Gleason grade. A Gleason grading algorithm would be pertinent and useful and can be applied to histology as a means to reduce workload or standardize pathological studies; however, in image space, it is difficult to make an accurate grade prediction. The pathologists included in our study disagreed most often along the G3 versus G4 boundary, and as a result, a predictive algorithm trained to predict Gleason grade would be heavily pathologist dependent. Predicting epithelium density is less pathologist dependent, where the ground truth is determined based on a morphological algorithm and only the lesion boundaries vary. Additionally, predicting a histological feature allows for a continuous risk scale, ultimately leaving the judgment call up to the radiologist reading the image.

In our previously published study demonstrating RPM, we included a subanalysis demonstrating that model performance improved as more annotated lesions were added. In this study, performance plateaued when about 360 total lesions had been included in the training set.^19^ In this study, there were three observers in the MP dataset that marked less than 360 total lesions that met our size criteria. It stands to reason that if the training set was expanded with the addition of more samples, the algorithm’s performance may continue to improve. This may be an avenue for future research.

Literature values for the diagnostic power of ADC vary depending on the study and the conditions under which the analysis was performed. Our group has previously published a more in-depth analysis of ROCs on ADC,^20^ reporting an AUC of 0.799 for discriminating high-grade lesions from low-grade and normal tissue. Other studies have reported an AUC of 0.81^34^ or as high as 0.9.^35^^,^^36^ The result of an ROC is highly dependent on the dataset and the postprocessing decisions made by the investigators. Prior studies evaluated using a radiologist-drawn index lesion will report a higher AUC than studies including more and smaller ROIs. While a direct comparison between the AUCs reported here and other studies may not be useful, we believe that a statistical comparison between ADC and our proposed method demonstrates additional added value to that currently clinically available despite a lower AUC.

There are several areas for improvement that future studies may address. The use of unlabeled consensus lesions rather than specifically denoted benign atrophy or healthy tissue is a confounding variable. Additionally, noncancerous pathologies (benign prostatic hyperplasia and prostatitis) are not explicitly labeled and thus may be present in the dataset. The technique used in this study requires ADC maps made with higher b-values than are normally clinically collected. There is evidence that high b-value scans may not distinguish cancer more effectively than what is currently used clinically;^37^ RPM algorithms trained using lower b-value scans may be as effective but more easily translatable into the current clinical workflow. More research is necessary to determine which pulse sequences and which MRI parameters result in the best RPM schema. This technique was previously published on a cohort of 39 patients and that cohort was included in this study. Future studies should validate this technique on externally acquired data.

In conclusion, this study demonstrates that radiopathomic maps of epithelium density derived from annotations performed by different pathologists distinguish high-grade prostate cancer from G3 and benign atrophy. We found substantial lesion-wise variability between our pathologists. The pathologist-specific RPM models produced statistically different mean epithelium density values; however, if these models are combined into a consensus model, the consensus model outperforms ADC and produces high contrast clusters in locations of high-grade tumor. These maps may be useful to augment image-guided biopsies or radiation treatment in the future.
